# Beyond traditional dairy veterinary services: ‘It's not just about the cows!’

**DOI:** 10.4102/jsava.v86i1.1221

**Published:** 2015-05-28

**Authors:** Martin L. van der Leek

**Affiliations:** 1 Department of Production Animal Studies, University of Pretoria, South Africa

## Abstract

It remains a challenge for the role of the dairy veterinarian to move beyond that traditionally held. In larger herds with a high reproductive workload, we are at great risk of becoming specialist technicians. Instead we seek greater involvement, to deliver comprehensive services and to be recognised for them, personally and financially. Given the frequency of our visits, knowledge and analytical skills we are in a unique position to provide inputs that complement advice given by other consultants. Failure to do so has economic consequences for both veterinarian and dairyman. The opportunity for and value of inputs will differ for every client, and we need to remain cognizant of their motivation. This review article shares perspectives, opportunities and tools that might enable moving beyond the traditional role. It starts with a review of available research describing the dynamic between dairyman and veterinarian and how this might impact an animal health production management programme. A description of the experiences of others follows, interspersed by the personal experiences of the author, working with large total mixed ration-fed herds in the United States of America. The following attributes and roles can be associated with a significant economic impact: gatekeeper; conduit; executor; verifier; monitor; facilitator and mediator; trainer, motivator and coach; applied nutritionist; technologist; champion of animal welfare, food safety and judicious antibiotic use; and confidant. Each is elucidated and described in context, revealing a need for continuing education. The nature of the relationship between veterinarian and client will determine the opportunity for and value of each. The veterinarian is in a unique position to become an integral part of the management team and to be fairly compensated as such. The onus rests on the veterinarian to broaden his/her knowledge and skills and to demonstrate their value.

## Introduction

Herd health programmes describe different combinations of services, but the author prefers the use of animal health and production management (AHPM), as described by Gay (2014). The author recognises, however, that the term ‘herd health’ is entrenched in the vernacular and might be used to describe any of the variants. AHPM programmes go beyond those that typically include reproductive services and the control of disease (clinical and subclinical). The use of the singular ‘animal’ acknowledges the importance of the individual animal as a component of the herd. It is the challenge of the AHPM veterinarian to optimise the biological functioning of the individual animal, the herd and by extension the factors that influence these, to include several of a seemingly non-veterinary nature:

Milk production is under constant economical, societal, and environmental challenges, which constrains dairy farmers responding to the increasing demands of a growing world population for a wholesome and economical milk supply. To meet these challenges dairy farmers must continuously adapt their milk production systems by relying on specialists to provide guidelines. Dairy production medicine integrates veterinary medicine and animal science into a system to produce milk profitably. The design, implementation, and management of this system is multidisciplinary, including clinical medicine, economics, epidemiology, food safety, genetics, human resource management, nutrition, preventive medicine, and reproduction. To be profitable without neglecting animal welfare and food safety, these specialties must work in concert to harmonize management. (Risco & Melendez 2011:ix)

A pertinent question is how to derive additional income from doing so. Primarily the need for AHPM programmes may be driven by the farmer, who is trying to increase production and/or decrease costs. An alternative model is one driven by the processor, retailer, consumer and/or regulator, where animal welfare, food safety and antibiotic resistance provide the momentum and income opportunity. Although they may be alluded to, it is not the purpose of this article to provide checklists, standard operating procedures (SOPs) or benchmarks. Rather, it examines the framework and context of an AHPM programme to identify and share those considerations of a less technical nature that might add value and lead to greater job satisfaction.

## The literature

An excellent review of the herd health concept, the players and how the field has evolved over the years is provided by Gay (2014). The topic has also been addressed by De Kruif and Opsomer (2004), Ferguson (2004), Fetrow, Cady and Jones (2004) and in texts by Risco and Melendez (2011) and Green and Bradley (2012). Individual components, their execution, management and the value thereof are well established. Recent advances in disease prevention are summarised by LeBlanc *et al.* (2006).

Evaluating AHPM programmes in the field is a challenge, but dairying in The Netherlands allows for that. Unless otherwise noted, the reviewed studies were conducted there. South Africa had 2083 dairy operations in 2013. In 2012 these operations had an average of 293 cows and an average milk production per cow of 20.1 litres per day (Milk Producers Organisation 2013). In comparison, in 2012/2013 there were 18 600 dairy operations in The Netherlands, with an average of 88 cows and an average milk production per cow of 27.0 litres per day (Veepro 2014). In South Africa there are several milk processors, whereas in The Netherlands milk is marketed almost exclusively by FrieslandCampina (2014), a cooperative with 14 132 member dairy farms at year-end 2012 in The Netherlands, Germany and Belgium.

The largest dataset reported in the Dutch studies cited contained a total of 3986 herds. For these the average herd size was 85 (ranging from 34 to 464) and the average production was 8440 kg of milk per cow per year (or 27.7 litres per cow per day). Access to these data was granted by CRV BV (2014), a cattle improvement cooperative with about 27 000 dairy and beef farmers in The Netherlands and Flanders. This offers a large multisite database with some uniformity in the methods of data collection, but also implies greater uniformity given the strict production management guidelines enforced by FrieslandCampina. In South Africa there are multiple milk processors and no unified requirement that influences in any meaningful way how milk is produced.

### The need

In their paper ‘Major advances in disease prevention in dairy cattle’ LeBlanc *et al.* (2006) came to the following conclusion:

There is an ongoing challenge for prevention of many diseases; although there is still much to learn, information already exists to substantially reduce or prevent the disease altogether—the challenge is in effectively and consistently implementing the required management practices. Ever better understanding of epidemiology and pathophysiology will not in itself reduce the incidence of disease. The ability to translate emerging knowledge into on-farm application and actual prevention of problems requires understanding of the farm as an integrated system, a major component of which is educating and motivating humans to implement well designed practices. Understanding and accomplishing this final major step in the disease prevention process is both an advance and an ongoing challenge. (pp. 1276–1277)

Cannas da Silva *et al.* (2006a, 2006b) described the increasing concern about animal welfare, traceability, the safety of products of animal origin and drug resistance driving additional governmental legislation that in turn places the farmer under pressure. Besides clinical work, the farmer will also need support in efficacy, management, welfare, profitability, nutrition, prophylaxis, economics, reproduction, environmental protection, grassland management and so on. They recognise the need for the veterinarian to have the ability to evolve with the industry, possibly using a SWOT analysis: strengths, weaknesses, opportunities and trends. They recognise that other professionals have seized opportunities where veterinarians might have made a sizeable contribution (such as nutritionists, animal production engineers, biologists, etc.), and that we lack a background in psychology and communication skills. They summarise the situation as follows (Cannas da Silva *et al.* 2010):

The need for more prophylaxis and less therapy; Participate in housing design and remodelling; Comprehensive reproduction programmes and defined reproductive strategies; Acquire nutrition expertise; Control milk quality (SCC, yield, butterfat, protein); Provide consultancy (Farm progress and development); Show more concern and be involved in management issues and Acquire a knowledge of economics (advice, decision making). (p. 8)

As veterinary input evolves there is another shift under way that is driven by animal welfare, food safety and drug resistance. Veterinarians will increasingly be involved with standardised certification programmes. FrieslandCampina maintains milk quality and consumer trust by demanding that their membership operates within strict guidelines (including milk quality, salmonella control and antibiotic use programmes). There is a similar trend in the United States of America, with several farm certification programmes, including the National Dairy FARM Program (2014), Validus (2014) and FIVE-STAR^SM^ (2014). These remain voluntary and are sponsored by different segments of the industry, each with their own bias. Such programmes are yet to significantly affect how dairymen produce milk and veterinarians earn income, but the programmes of the Dairy Standard Agency (2015) and SA Livestock G.A.P. (2015) are gaining traction in South Africa.

### Perception, attitude and communication

Valeeva, Lam and Hogeveen (2007) examined the motivation of farmers to improve their mastitis management, the impact of incentives and any linkage between the two. For these farmers their individual farm performance and personal motivation exceeded any external motivation. Non-monetary factors were as important as the financial ones, and included farmer well-being and the satisfaction associated with good animal stewardship. Quality penalties were found to be an effective motivator. Interestingly, farmers could be grouped into one of three groups according to their motivation: premium or penalty motivated; motivated to have an efficient, well-organised farm that could readily comply with regulatory requirements; and those motivated purely by economics.

Klerkx and Jansen (2010) studied advisory services, including those for mastitis prevention, and found that advisory effectiveness depended on an adequate mix of, and balance between, push and pull measures. Push measures included the opportunity for advisors to improve their conveyance skills and to better coordinate theory and practice. Pull measures included raising farmer awareness and demand either directly or through financial incentives.

A Danish study (Kristensen & Enevoldsen 2008) was able to group farmers’ expectation when participating in a herd health programme into four categories: teamwork, animal welfare, knowledge dissemination and production. In contrast, their veterinarians believed that they were motivated only by production and profit, a glaring disconnect. When examining the role of owner behaviour and/or attitude on mastitis incidence, Jansen *et al.* (2009) found that owner attitude could best explain the variance in the variables measured. Identified as important were the farmers’ normative frames of reference, their perceptions about mastitis control and their appreciation of the negative consequences.

Studying udder health, Jansen, Renes and Lam (2010) examined the effectiveness of each of two communication strategies. The first was a typical informational and training strategy (including brochures, SOPs, and software), whilst the second focused solely on increasing the adoption of wearing gloves during milking. They concluded that both strategies were effective and could be used in conjunction for greater benefit. However, a farmer's motivation and the aim of the communication should influence which was predominantly used.

In a Danish study Kristensen and Jakobsen (2011) explored the commonly held belief that clients are generally irrational when they do not follow what veterinarians would consider to be substantive advice. Instead they concluded that this behaviour has a psychological basis, underpinned by a natural reluctance to react to a suggested change in behaviour. There needs to be an awareness of this issue and the techniques needed to communicate effectively. They stressed the importance of appreciating the context in which the farmer might be evaluating any advice and then acting upon it.

### Participation and value

A survey evaluating the perception of dairy farmers regarding their veterinary herd health management (VHHM) plans found that farms with a plan had better herd performance and that the farmers were generally satisfied (Derks *et al.* 2012). Of note was that goal setting and re-evaluation were not always part of the plan, and that off-farm consultation was often not charged for and/or not specified on the bill. The results illustrated a lack of communication and/or product differentiation. Satisfaction with their programme was associated with a lower calving interval and bulk tank somatic cell count (SCC) but not 305-day milk production.

Derks *et al.* (2013a) set out to determine how many farmers participate in VHHM programmes, how the VHHM plan was executed and if farmer attitude was related to their participation. There was a 68.6% participation in VHHM, with fertility checks and reproduction advice ranking highest and housing and claw health ranking lowest. There were associations between farmer attitude, the value they attached to the advice and their level of participation in VHHM programmes, but it was concluded that this required further study.

A survey of 1913 dairy farmers found that those who participated in VHHM plans produced 336 kg of milk/cow more per year and their SCC was 8340 cells/mL lower. There were, however, negative associations with reproduction and culling. The economic benefit of a VHHM programme and its longevity is difficult to measure. There were many challenges and confounders: causality could not be examined; the response rate was poor; the quality of the veterinary advice was unknown; it was possible that poor communication and understanding existed between veterinarian and farmer; variable farmer compliance was noted and an increased need for advice might have been precipitated by problems (Derks *et al.* 2014).

The perception of the veterinarian regarding the dairyman and in turn the dairyman's perception of the veterinarian as it related to participation, the advantages and disadvantages and the reasons why dairymen quit AHPM programmes, have been examined (Lievaart & Noordhuizen 1999; Lievaart *et al.* 1999).

Comparative herd health participation rates were 37.2% in The Netherlands (Lievaart & Noordhuizen 1999), whereas in the UK 40% of practices had less than 25% of their herds involved, whilst 30% of practices had more than 50% of their herds involved (Hall & Wapenaar 2012).

#### The experience

In a well-received editorial, Guterbock (2001) shared from personal experience a different perspective – that of the dairyman. It was only after being employed by and then becoming part owner of a dairy that his view crystallised. This editorial was widely read, contributed valuable insight and explained why AHPM programmes might be embraced with so little enthusiasm. The author takes the liberty of summarising his editorial in the following paragraphs.

**Running a large dairy involves more than managing cows:** The complexity of running a large dairy business means that cow care is delegated to employees, whilst the owner is preoccupied with employee management, feed purchases, manure management, credit lines, inspectors/regulators, tax considerations and possibly a farming enterprise. This creates an opportunity for the veterinarian to observe and monitor many of the animal-related activities on the farm, and we should leverage this advantage. As newer and possibly more important issues arise, animal management areas might be pushed lower down the list (typically herd replacements and dry cows), even if not by intent. It is worth noting that there is an inherent and important difference in risk taking between the dairyman and the veterinarian. Dairymen seem willing to make what seem to be quick and incomplete decisions. We need to remain cognizant of the number of decisions that they have to make and the priority that they assign to each. Enlightened owners and managers recognise these shortcomings plus the need for professional advice, and are willing to pay for outside services.

**Veterinary emergencies are not really emergencies:** The bulk of the veterinarian's work affects only one animal at a time: a dystocia, a left displaced abomasum (LDA) or a sick calf. It is no surprise then that an owner and employees might be less than interested in a lecture about an individual case. Given the scope and complexity of the operation, these events are of relatively minor importance. True emergencies are those that affect all the cows, and feeding and milking top that list. A faulty milk-cooling system, a broken vacuum pump and a disabled mixer-feeder are examples. Suffice to say that a good mechanic and devoted milkers are more important than a herd veterinarian. When the cows are not presented in a timely fashion for reproductive examinations we need to appreciate that there might have been a ‘real’ emergency.

**Veterinarians need to stop thinking about per-cow averages:** Veterinary training focuses on the individual animal and only on basic epidemiological principles. We often use simplified statistics such as the average of a group. Besides the inherent deficiencies, including lag an average does not detect those individual animals that might respond to an intervention. Guterbock gave examples of meaningful interventions:

- Monitoring fresh cows with low daily milk instead of peak milk.
- Monitoring cows not bred instead of average days open.
- Blood sampling for serum total protein instead of calf death rate.
- Monitoring heifers not bred by 15 months of age instead of the age of first calving.

**Most traditional veterinary tasks are not performed by veterinarians:** On large farms all routine veterinary work cannot be done, and even less so the more clients are added. The owner delegates work to a manager who in turn delegates it to employees. Veterinarians too then have to delegate work, but need to do so in such a way that they have and keep a vested interest through training and monitoring. Giving up mundane veterinary tasks need not instil fear that we are losing or giving away our profession.

**Cases veterinarians brag about generally have bad outcomes:** An understanding and appreciation of the relative economics of veterinary work is enlightening. The satisfaction of repairing an LDA or removing a calf by foetotomy cannot be disputed, but the consequence is often less impressive. The cow with an LDA might also be suffering from ketosis and the cow with dystocia might already have nerve damage. In both cases the cows would best have been sent to slaughter. Instead we should get involved with the transition programme and help the breeders to avoid breeding animals that have a frame size that is too small.

**Giving advice is easier than receiving it:** Veterinarians need to understand the importance of the perceived value of their advice, its timing and why it might not be accepted. Sometimes the owner might not agree that it is important, may find its implementation too disruptive, does not want to confront the issue or might simply elect to ignore it. They might not even have been aware of the need or be too ashamed to acknowledge that they had known about it for some time. Dairymen generally do not have the time or the interest to spend studying their computerised record systems, yet they are often aware of the issues without having to consult them. Overloading management with concerns based on the extensive evaluation of records and their needed interventions might show off our analytical skills, but add little but confusion when these are not prioritised. We also need to appreciate that problems are not always the consequence of bad management but rather the result of conditions not under their control. These might include temporary overcrowding because of the calving cycle or market issues affecting the availability of feeds. In reality the biological variation and complexity of the dairy business almost guarantees that systems and/or animals will fail. The astute dairyman is one who anticipates such events and minimises their impact. Their inability to be perfect should not be met with disdain, but with understanding.

**Veterinarians are not the only smart people out there:** Veterinarians might have above-average intelligence but they are not the sole advisors on the well-being of the clients’ herds. There are many others that influence them and add value: fellow producers, nutritionists, company technical staff, etc. We need to be cognizant of their roles and strive to become an integral part of a management team, utilising our skills to add value to the team and to the dairyman.

Guterbock summarised by indicating that a stand-alone and outside consultant, even if present weekly, will never have the same impact as a local practitioner with a vested interest in the operation, who is knowledgeable about the farm, visits regularly (sometimes unannounced) and is an accepted part of the management team.

In the following paragraphs, the author shares his career evolution as experienced in the USA. Although its relevance might be questioned because it was on another continent, the themes are universal and driven by an increase in herd size.

**Traditional practice:** The author first practised as a solo farm-animal veterinarian in Florida. Dairy comprised 50% of the practice with 10 herds and an average herd size of 500 milking cows (concentrate- plus hay-fed herds). This was a traditional practice with reproductive and sick cow work. Emergency calls for sick animals or obstetric cases were the order of the day. Typical herd health advice was sought on issues such as mastitis and calf diarrhoea.

**Technician:** There followed a solo dairy practice in New Mexico with 10 clients and an average of 2000 milking cows per herd (total mixed ration [TMR] corn silage-fed herds). The work was almost exclusively of a routine technical nature and included rectal palpations, displaced abomasum (DA) surgeries and the vaccination of calves against brucellosis. In excess of 2000 cows were palpated per week, and over one 3-year period there were more than 2000 DA surgeries. The opportunity for consultation increased steadily over the 6 years in practice and included the writing of SOPs, the training of employees, the programming of herd management software, data analysis and applied nutrition. The large number of DAs provided the impetus to better understand nutrition. Obtaining an early copy of the Cornell-Penn-Miner Dairy model ration balancing software (CPM Dairy 2014), he requested client rations and engaged in a dialogue with nutritionists. This self-education phase resulted in no additional income, and ironically the practice lost substantial income as the DA problem was resolved. It did, however, engender a huge amount of respect, and substantive income was subsequently generated by this activity. As an aside and of note was that all employees were of Hispanic origin, necessitating a command of Spanish.

**Dairy manager:** The technical and physical nature of dairy practice enticed the author to accept the position of in-house veterinarian and consultant employed by one of his clients. As in the case of Guterbock (2001), it allowed an appreciation of the dairy business in its totality and the relatively minor importance of the veterinarian's role. This was a humbling experience. Specific lessons learnt included the importance of execution; employee structure, training and coaching; and applied nutrition. There followed an opportunity to join a progressive dairy cooperative that already had several veterinarians on staff either as employees or part owners. These included Walt Guterbock and Gordie Jones, well respected AHPM veterinarians that had moved from practice to employed consultant to part dairy owners. The author managed dairies ranging in size from 2300 to 5500 milking cows, including a 3000 cow start-up herd. The 5500 milking cow herd was milked three times per day in two double 40 parlours, had 12 000 animals on-site and approximately 80 employees.

**Private consultant:** A consultant would visit a dairy on a regular basis, typically for a day, provide recommendations and leave. At best they might do some initial training. The author elected instead to offer services on a project basis. By remaining on site it was possible to facilitate execution, identify and structure employees for the task, train these employees, modify the recommendations as needed and institute a monitoring system. Examples of such projects included the management of a dairy in bankruptcy on behalf of the lender (a 3-month project) and the conversion of a 3000 cow dairy (6000 animals on-site) to electronic identification (a 3-week project).

## Core concepts

### Variation

Inherently there is a sizable amount of variation in what is a complex biological system, and this variation can increase markedly as herd size increases. Sources of variation might include employees treating cows differently for the same disease; following different milking procedures; feed purchased from a less reputable feed mill with fluctuations in quality; differences in grain processing; inconsistent mixing of the ration (Sova *et al.* 2014), and many more. There is a huge opportunity in identifying these, prioritising them and then systematically addressing the variation. An awareness of what might be considered acceptable variation and the implication thereof holds value. For example, the inherent variation in daily milk production requires 350 cows per group in a completely randomised design to detect a meaningful difference of 1 kg/day per treatment (Yandell 1997).

### Bottlenecks

Bottlenecks are rate-limiting steps that hold the dairy back from achieving its full potential in spite of excellence in other areas. Credit for the first application of this concept to the management of large dairy operations goes to Fetrow (2009) and Jones (2012) also made extensive use of it. A good example is a ‘broken’ transition programme. Neither the best milk cow rations nor perfect cow comfort can compensate for a transition period where the cows suffer from fat cow syndrome. Initially the number of causes for variation and bottlenecks might seem overwhelming and difficult to prioritise. An intimate working knowledge of the dairy operation is critical to prioritise the needs and determine the order of attack. The veterinarian should resist – as hard as it might be – addressing too many issues at one time, overwhelming both the dairyman and employees.

## Context

It is essential that the client's decision making, or lack thereof, be valued in the context in which the decisions are being made. The veterinarian needs to be aware of the nature of the business and the financial drivers: ‘The practicing veterinarian's ability to translate knowledge into on-farm application requires a profound understanding of a dairy farm as an integrated system’ (Kristensen & Jakobsen 2011).

The demands and personal resources available are quite different when comparing a farmer who is just dairying versus a farmer who is also doing his own cropping, or one that is in the middle of an expansion. Cost-savings often entice dairymen to take on endeavours that remove them from the daily animal activities. Many dairymen have lamented the fact that they were no longer able to be in the parlour every day when the hospital cows were being treated, but instead were preoccupied with manure management and fertiliser decisions. Typically for a TMR herd in the USA the owner can actively be involved with daily operations and animal activities as long as the number of milking cows remains below 1500.

The financial underpinnings of the dairy operation affect the attitude and behaviour of the farmer. Scenarios include an inexperienced first-generation farmer with a high debt load; an experienced farmer with a rapidly expanding herd; a third-generation farmer with a sizeable inheritance; a dairy that is just a small part of a farming enterprise being operated for tax purposes not profit; or the farmer that by nature is a low-input dairyman. Each of these implies differing levels of interest in an AHPM programme and the extent thereof. It can, however, be quite frustrating when dairymen seemingly ignore what might seem an opportune time for an expanded AHPM programme. Servicing debt and cash flow can be powerful motivators for decision making that might seem counter-intuitive on the face of it. A typical example is moderate overcrowding where the additional cows do not add to the overhead expense but add milk and cash flow.

Culling parameters also need to be appreciated in context. As milk production per cow increases and reproduction is excellent, the milk production of cows destined for culling might increase to levels that make culling seem counter-intuitive. However, the availability of heifers that will push poorer-producing animals out of the herd reflects good management, and allows for cull cows to be sold as productive animals instead of slaughter. It is also the convention that a lower cull rate is associated with good management, but the exception is an important one. In herds with excellent reproduction there will be an excess of heifers. These can either be sold or used to replace older and lower- producing cows. Consequently the herd cull rate could legitimately be higher, the bulk of these being voluntary culls.

### Communication and people skills

A phrase shared with medical students is the ‘4 A's for success’, which are availability, affability, affordability and ability (Tonkin 2002), and specifically in that order. It serves to underscore that ability only comes into play after the others have been considered by a client. Unfortunately veterinarians do not receive adequate training in communication and people skills. The lack of these is amplified on a dairy where we have to deal with an owner, manager/s and employees, each necessitating a slightly different approach.

As a profession we tend to be confident, sometimes overly so, as was suggested by Derks *et al.* (2013b) when they showed a marked disconnect in communication between veterinarians and their dairy farmer clients. Veterinarians either made assumptions about the need for herd health management and goal setting and/or misread the relative importance of their clients’ needs. Veterinarians did not actively communicate to clarify these differences. Farmers in turn do not readily volunteer such information, illustrating the need to skilfully elicit it.

As with the style of communication, it is equally important to take into consideration the culture, literacy, training and skill level of the target audience, be it the dairyman and/or employees (Arcury, Estrada & Quandt 2010; Estrada 2003a). As with other health professionals, veterinarians tend to be overly detailed and complicated, as well as separated from the conditions and environment in which the audience performs their tasks. A good example that is often struggled with is in the milking parlour when attempting to convey the difference between cleanliness and sterility.

## The opportunities

The author will take a somewhat different approach from the typical description of the components of an AHPM programme, instead sharing another perspective. When developing and showcasing these skills one cannot expect to generate the income we might ultimately expect. This can be both a frustrating and a rewarding time. The following describes attributes and roles that although appearing subjective can be associated with a fundamental role in the dairy business and have a significant economic impact: gatekeeper; conduit; executor; verifier; monitor; facilitator and mediator; trainer, motivator and coach; applied nutritionist; technologist; champion of animal welfare, food safety and judicious antibiotic use; confidant.

### Gatekeeper

Dairymen are continually presented with new products, technologies and procedures. They do not always have the knowledge or the time to evaluate the merits of these. Instead they rely on salespeople and/or the technical staff of the presenting company to guide their decision. But for the rare exceptions, companies are reputable and the science behind the offer is sound. The question is not the scientific validity of the offer but whether it has a positive benefit-cost ratio for a particular dairy. As veterinarians we are in a unique position to evaluate such offers and play the role of gatekeeper. A sound knowledge of partial budgeting is useful and so is the need for accurate and detailed production information. It is the author's experience that once accepted, such additions are rarely re-evaluated unless the dairy is under considerable financial pressure. Often promoted, on-farm trials in small herds are a challenge to conduct since statistically valid results generally require more than 300 cows per treatment group (Yandell 1997). The opportunity and value of influencing and impacting that initial decision is easily underestimated.

### Conduit

Given our knowledge and analytical skills and the frequency of our visits, veterinarians can provide inputs that complement the advice given by other consultants. Any professional that invites another for consultation engenders respect in the eyes of the client. These need not be limited to consultants from within the profession. Guterbock (2001) stated succinctly that ‘We aren’t the only smart people out there’ and so did Richard Patton (2014) when he wrote in his blog: ‘The truth can come from anywhere (even a veterinarian!).’ Reputable outside consultants will further the dairyman's business and validate our influence. It is also true that familiarity breeds contempt, and clients do not always respond to valuable advice even when delivered repeatedly. The novelty of that same advice uttered by an outside consultant is an eloquent way to nudge a client into action.

### Executor

Advice, training and SOPs are of little value if executed poorly. This reflects negatively on veterinarians and their skills, even if it was not their direct responsibility. Both parties are now dissatisfied and the perceived value of our advice is cheapened in its totality. Since we have a vested interest, it is in our best interest to assume control over the execution of an SOP. This might require multiple visits and some at inopportune times. Although we will initially be reluctant to bill for this, the goal is to demonstrate value, with the expectation that future SOPs would generate fees for writing them, for training employees, for execution and then for monitoring.

### Verifier

On large dairies the collection and entry of data becomes a tedious task. Not only is the volume of data entry an issue, but so too the fact that more than one individual might be involved in data capture and entry. It is risky to give advice that relies on data without confirming their veracity. There might be errors in animal identification (wrong animal), errors in data entry (wrong animal, wrong event), inadequate or wrong case definitions, wrong diagnoses and/or wrong formulae. Data management has been simplified by the availability of cow-side versions of the common herd management software programmes and individual animal electronic identification, but errors are never completely eliminated (garbage in, garbage out).

Errors are not restricted to the management of data, but also occur during the execution of what may seem like simple tasks. With large numbers of animals, time constraints and the repetitive nature of some of these tasks, error rates can be high. Multi-step procedures are especially prone to error. Treatment of a wrong quarter and a missed synchronisation step are examples. Procedural (or practical) drift is a theory defined by Snook (2000) as the ‘slow uncoupling of practice from procedure’. Typically and over time strict adherence to a procedure fades, and this is best identified before it reaches a critical point.

### Monitor

The monitoring of herd production and disease parameters is a classic component of AHPM. Statistics may have been generated by the herd management software or be calculated from raw data. A working knowledge of herd management software programs is essential, again implying a huge initial investment and steep learning curve. Serious consideration should be given to the veterinarian acquiring copies of the software programs used by their clientele.

There are important caveats when considering monthly monitoring. Firstly, the number of parameters monitored and presented should fit on no more than a single page. Although acceptable to monitor multiple statistics, it is important not to induce ‘monitor fatigue’ in the client by presenting pages of data. It does not matter how pretty and colourful the graphs and tables are. Secondly, the items monitored may have to be repackaged to better fit the perspective and needs of the dairyman. Pertinent examples include milk production expressed as the amount of milk shipped per day versus the classic milk production per cow per day (the former being a better reflection of cash flow), and fertility expressed as the number of cows pregnant per month instead of a pregnancy rate (the former being a better indicator of the future herd profile). As described by Guterbock (2001), there is a need to avoid the trap created by averages, given the concerns of momentum, lag and bias. Instead we should focus on the variation in the data, finding those animals that would benefit from an intervention. The average number of days in a close-up pen is a valuable metric, but it is far more important to detect animals that are either in this pen for too many days (and on an expensive ration) or too short a period of time (lack of anionic diet effect). The author refers to this approach as ‘monitor averages, manage outliers’. Valuable tools for monitoring variation are statistical process control (Risco, De Vries & Thatcher 2007) and cohort analysis (Smith 2012).

Besides data it is also essential to monitor the immediate success or failure of a new procedure and its execution, both on a short- and a long-term basis. Procedural drift (Snook 2000) is best not ameliorated by tighter rules and regulation, but rather by a combination of techniques that might include regular retraining and acknowledgement of it (e.g. by certification), an understanding of the consequences of protocol failure, allowing ownership in the process, positive or negative incentives and the fostering of relationships between those involved in the procedure (Carillo 2013).

### Facilitator and mediator

Some degree of tension and/or animosity is the norm between owner and manager, manager and employees. The veterinarian, as a neutral outsider, can establish a rapport with employees and gain their confidence, revealing issues that would not be identified otherwise: an employee afraid to share that they do not really understand what is expected of them or one aware of wrongdoing but fearing reprisal if reported. An all too typical and often valid complaint is that of an owner unnecessarily meddling in daily activities or regularly modifying procedures. The veterinarian is in a unique position to raise such issues and mediate any ensuing discussions. Sadly, veterinarians are largely unprepared for this role, having had no training in negotiation, facilitation and mediation. Care needs to be exercised in managing these relationships and divulging the information should be balanced with a veterinarian's typical desire to intervene immediately. A short-term fix at the expense of an important insider relationship is unwise; the difference between: ‘the calf guys have no idea what they are doing’ versus ‘I think that I can help the calf guys do a better job.’

It also implies that one is fluent in all of the languages spoken on the farm, as this engenders a huge amount of respect. The author concedes that the multiple languages often spoken on a South African dairy offers a unique challenge. Without the language skills the veterinarian remains removed and less effective. Although fluent in ‘dairy Spanish’, accurate technical language and cultural nuances remained an issue for the author. A breakthrough was teaming up with a Hispanic leadership coach and mediator with extensive dairy experience to bridge the cultural and literacy gap, to reinforce and verify technical training, to provide employee feedback and to mediate differences of opinion (Arcury *et al.* 2010; Estrada 2003a). Regular and routine visits were eagerly anticipated by employees as he was seen to further their best interests.

### Trainer, motivator and coach

The technical training of employees might seem an easy and obvious opportunity, cognizant of potential language and cultural issues. The added value when managing SOP execution has been discussed, but one should not forget the opportunity to motivate and build employee confidence (Estrada 2010). Employees should be empowered by sharing the basic knowledge of management, biology and disease and how they personally play an integral role in the well-being of the cows and the dairy. Employees are an important extension of the veterinarian's presence on the farm, and one that should not be feared because they might perform some of the duties traditionally performed by the veterinarian. This requires the ability to connect and to transfer information at a level understood by the audience. Working with the employees allows the veterinarian to identify those who grasp the necessary concepts, those who have leadership abilities and/or those who should work under direct supervision. In concert with the dairyman, the veterinarian is in a position to assist in developing the best management structure, individual responsibilities and authorities (Estrada 2003b; Estrada & Simmonds 2005; Simmonds & Estrada 2004).

### Applied nutritionist

One might easily argue that this topic should be at the top of the list since it is the biggest expense, drives production and has a major effect on immunity and health (Ingvartsen & Moyes 2013). Indeed, the need for veterinary services decreases exponentially as nutrition is optimised. Perceived as primarily the role of the nutritionist, the roles of veterinarian and nutritionist need not clash, in spite of an all too common animosity. The veterinarian should be familiar with ration balancing software and printouts and can assist in monitoring feed quality, TMR mixing, feedbunk management and manure quality. Individually neither veterinarians nor nutritionists spend appreciable amounts of time on the dairy, but by working together they double the opportunity to make observations and detect variation and bottlenecks. By actively following nutritional changes and variability the veterinarian can start linking these with changes in animal production and health.

Conceptually it is useful to consider that there are five rations on a dairy and that it might be useful to evaluate the feeding system in this way:

- Ration as formulated: is it appropriate for the production group, are feed analyses or book values used, and is the ration balancing software based on the Cornell Net Carbohydrate and Protein System or a similar model (Offner & Sauvant 2004)?
- Ration as mixed: Are the correct ingredients, amounts and order of loading used?
- Ration as delivered: Is the fibre adequately processed and is the correct amount delivered per pen?
- Ration as consumed by each cow: Is there sufficient bunk space; is there sorting?
- Ration that is digested: What is the protein quality, the starch availability, and are there nutrients excreted in the manure?

Manure consistency and content are not only indicators of disease, but also reflect ration quality. Overly stiff manure may suggest a lack of fermentable carbohydrate or the feeding of poor-quality forage, both offering an opportunity to improve the ration and increase milk production. Such observations communicated to the nutritionist might allow ration modification and/or intervention days ahead of the next nutritionist visit. Observing the mixing of the TMR might suggest ingredient amounts rounded off, an inconsistent ingredient loading order, insufficient time for processing and mixing and/or blades in need of sharpening or replacing. Feed management and shrink is an underestimated cause of significant financial loss. It is generally accepted that feeds and forages when visibly scattered around the feed area account for at least 10% loss or shrink (Harner *et al.* 2011; Mikus 2012).

The veterinarian should appreciate and motivate that feeds be analysed instead of using book values and that state of the art ration balancing software be used, such as CPM Dairy (2014) and Agricultural Modeling and Training Systems (AMTS, 2014). These model the microbial output of the rumen, intestinal digestion and balance rations on the amino acid and fatty acid level. This degree of sophistication optimises the cost of a ration in spite of feeding more expensive, higher-quality ingredients and reduces the excretion of unwanted levels of nutrients into the environment, including nitrogen and phosphorus. Recently adopted but not yet widely used in the dairy industry is the concept of feed efficiency (Hutjens 2012; Maulfair, Heinrichs & Ishler 2014), a good motivator for closer scrutiny of the feeding programme and the collection of accurate feed intake data.

### Technologist

One cannot ignore the ever-increasing role of technology, including electronic identification, automatic take-offs, in-line milk sampling (for conductivity, beta hydroxybutyrate, lactate dehydrogenase, milk urea nitrogen and progesterone), remote electronic scales, motion sensors for heat detection, rumen boluses (measuring body temperature, ruminations and/or pH), robotic calf feeders and robotic milkers. Despite obvious advantages, there are causes for concern. Firstly the owner, employees and/or veterinarian have to be trained on and become familiar with the new equipment, its functioning and the associated software. The learning curve can be steep and it is not uncommon that the owner relies on the employees. Without the necessary schooling and/or training, a financial investment might be used at only a fraction of its capability. Secondly, these systems are only of value if working properly and then doing so accurately. Expected to use a technology, lay employees easily lose respect for the equipment as well as the owner when they are left operating malfunctioning equipment. Thirdly, this equipment generates huge amounts of additional data that need evaluation and application. In short, technology does not work automatically and in a vacuum, but draws additional resources. Technology must be managed. The veterinarian is in a unique position to embrace an individual technology, monitor its use and assist in managing its data. Gaining familiarity with these things involves an investment in time with little additional income, but will engender respect, demonstrate added value and should generate income in the future.

### Champion of animal welfare, food safety and judicious antibiotic use

The initiative to champion animal welfare could come from the veterinarian, the farmer, or may come from outside (processor, retailer, end-user and/or regulator). The desire or need to decrease costs and/or undertake additional capital improvements may confound the veterinarian's interest in promoting animal welfare, especially when we might fear losing a client. The veterinarian can continue to emphasise the direct connection between good animal husbandry, cow comfort and optimised production, since these and animal welfare are not mutually exclusive. Veterinarians have championed the routine use of painkillers when performing procedures such as calf dehorning as well as the banning of tail docking. Industry has created certification programmes driven by consumer sentiment, but they might be biased since they are designed and enforced by industry themselves. Finally, there are standards and certification programmes imposed through regulation and enforced by legislation. Food safety and antibiotic residues go hand in hand with such programmes and so too the veterinarians’ traditional involvement in treatment SOPs, judicious drug use and adherence to appropriate drug withdrawal times. Increasingly the veterinarian will be utilised to certify dairies according to the requirements of these programmes.

### Confidant

This role is not the sole prerogative of the AHPM veterinarian, and many veterinarians have developed a relationship with a client where they are asked for advice regarding personal and business matters. Here the author refers to the case where the veterinarian is intimately involved in their client's dairy business, not only in executing an AHPM programme but also acting as a sounding board for their client's future ideas, plans and objectives. These might include the construction of new dairy, the purchase of additional animals, the planning of an on-site expansion or the purchase of additional farms. Although not necessarily having the expertise, this reflects the ultimate acknowledgement of the value of the veterinarian's unbiased analytical skills centred on the well-being of the cow.

## Conclusion

Globally herd sizes are increasing, with more work being performed in-house. The bulk of this work is of a technical nature and can be done as effectively by the employees as by a veterinarian at a fraction of the cost. This should not instil fear, but creates opportunity. Moving beyond traditional veterinary services requires the veterinarian to retrain and retool, with a positive economic outcome for both the veterinarian and dairyman. It is the case that the dairyman is often unaware of the value that might be added by their veterinarian. It is equally unfortunate when the veterinarian concludes that their client just does not grasp the concept of AHPM. A sustainable AHPM programme requires a shift in mind-set by both the veterinarian and client. The interested veterinarian needs to drive this change, even though it requires a huge investment and initially offers little in the way of financial gain.
